# A study on Taiwan’s vocational senior high school teachers’ teaching identity and teaching transformation when facing a new competency-based curriculum

**DOI:** 10.3389/fpsyg.2024.1290551

**Published:** 2024-01-31

**Authors:** Tzu-Chien Lin, Yi-Sang Lee, Jian-Hong Ye

**Affiliations:** ^1^Department of Industrial Education, National Taiwan Normal University, Taipei City, Taiwan; ^2^Faculty of Education, Beijing Normal University, Beijing, China; ^3^National Institute of Vocational Education, Beijing Normal University, Beijing, China

**Keywords:** competency-oriented teaching, sense of identity, teaching practice, teaching preparation, teaching transformation, technical and vocational education, implicit theory, teaching reform

## Abstract

**Introduction:**

The competency of education is advocated in the 2023 United Nations Sustainable Development Goals. UNESCO encourages countries to provide inclusive, equitable, competency education and lifelong learning opportunities for all. Starting from 2019, 12-Year-Basic-Education, a new curriculum, was fully implemented in Taiwan to conform to the competency education strategy. For the teachers on site, teachers’ understanding of the new curriculum and teaching practice has a direct impact on the competency of the implementation of this new policy. The main purpose of this study was to explore the relationship between vocational senior high school teachers’ competency-oriented teaching, teaching identity and teaching transformation regarding this new curriculum.

**Methods:**

In order to effectively expand teachers’ understanding of competency-oriented teaching identity, this study put forward 6 hypothetical approaches based on the implicit theory of teaching transformation. The 747 valid questionnaires accounted for 97.1% of the total recovered questionnaires. The reliability and validity analyses, as well as overall model fitting analysis and research model validation were performed on these valid questionnaires.

**Results:**

The results of the study showed: (1) With the background of Competency-oriented teaching, teachers’ teaching attitude and teaching willingness has a positive impact on teaching identity; (2) teachers’ teaching identity has a positive impact on teaching preparation, teaching practice, further study, three types of teaching transformation. In summary, three conclusions from this study were concluded on the aspects of teaching preparation, teaching practice and further study on practical competency-oriented teaching, teaching identity and teaching transformation in the educational field.

**Conclusion:**

Three conclusions were derived for the relationships among these six constructs: (1) Teachers with a “good attitude” and “strong willingness” to teach, a “high sense of identification” acceptance and full implement of “teaching preparation” are considered as teachers with a “foresight and a visionary predictive style”; (2) Teachers with a high sense of self-awareness who can fully practice “teaching practice” are teachers with a “pragmatic teaching by example style”; (3) Teachers with a “good attitude,” a “strong willingness” to teach, a “high sense of identification” acceptance and who are able to fully practice “advanced research” are considered as teachers with an “empowerment-enhancing coaching style”.

## 1 Introduction

Education can stimulate the practice of learning objectives of the Sustainable Development Goal. Competency-oriented teaching emphasizes cooperative learning and practice in life situations. Meanwhile, teachers should have diversified curriculum design and teaching capabilities, and should focus on cultivating students’ abilities and attitudes in life situations. To meet the needs of teaching diversity and to be adaptive, teacher evaluation should also be altered to fit the corresponding teaching evaluation (Luo, 2017). In line with the Quality Education Strategy (SDGs Progress Report, 2023), a new curriculum was implemented in 2019. Competency is “the knowledge, abilities and attitudes that a person should possess in order to adapt to current life and face future challenges” (Ministry of Education, 2020a). High-quality education programs are an important cornerstone for schools to improve teaching quality and enhance national competitiveness. Competency-oriented teaching focuses on students’ diverse exposure, independent learning, and interdisciplinary integration as for students should be treated as the main group in the learning process. Moreover, learning is a rolling adjustment and application capability accumulated from many different levels of educational situations and circumstances. The curriculum structure of competency-oriented teaching is obviously different from the traditional teaching which transfers knowledge from the teacher to the student and from the top to down teaching, Competency-oriented teaching makes teachers transformed from knowledge imparters to researchers, designers and implementers of core competency integrated courses (Huang and Tsai, 2015). Hence, teachers’ role and identity are important for competency-based courses.

“International Education 2.0” with the vision of “connecting with the world and linking the world” has been in practice in Taiwan since 2020 (Ministry of Education, 2020b). The composition of competency-oriented teaching is knowledge, attitude and skills (Ministry of Education, 2020a). When practicing the new curriculum at the teaching site, teachers are important promoters of competency (Tong and Lui, 2020). From the guidance teaching of traditional teaching to guided teaching, students can then develop the abilities of reading, explanation, thinking and integrated application (National Academy for Educational Research, 2014). Real-life dilemmas are complex, and the teaching design of core competency should not be limited to a single educational subject or field (Liu, 2020). In 2014, the Taiwan government announced the “Curriculum Guidelines of 12-Year Basic Education,” hereinafter referred to as the new curriculum, which was officially implemented in 2019, adaptive high-quality education was advocated, with the vision of “achieving every child—adaptive talent development and lifelong learning,” inspired students’ joy of life and self-confidence in life through adaptive education, advocated adaptive competency education and expanding international viewpoints. In 2021, the Organization for Economic Cooperation and Development (OECD) predicted that the function of schools in the future education field will continue to exist, but the learning patterns will face adjustment and changes, meaning that school learning is necessary, but school is not. More attention will be paid to the development of global digital and data technology management. Therefore, competency-oriented teaching, the recognition of teaching strategies and teaching transformation are important ability indicators that every learner or teacher must obtain in the future. Taking a comprehensive look at international education trends, international organizations such as the United Nations Educational, Scientific and Cultural Organization (UNESCO), the Organization for Economic Cooperation and Development (OECD), and the European Union (EU), scholars from various countries believe that traditional school education in the past only focused on instilling limited knowledge, which is no longer fit for future social changes and international trends. The importance of core competency and teaching transformation emphasizes that learning needs to be combined with life, and that there should be a continuous learning process from childhood training to lifelong learning. Since then, “core competency” has become an important concept leading educational innovation in various countries (OECD, 2016, 2018).

The purpose of this new 12-Year-Basic-Education curriculum in Taiwan is to strengthen the core competency teaching, to amend the past idea of focusing more on academic courses than on practical courses, neglecting intentional attitude, and promoting the vertical coherence and horizontal integration of the overall curriculum (National Academy for Educational Research, 2014). The new curriculum focuses on lifelong learners and also echoes the concept of spontaneity, interaction and common good that expands outwards layer by layer to form a continuously rolling round wheel image (Ministry of Education, 2014b). It emphasizes and represents the importance of each learning individual, encourages learners to interact and connect with the surrounding environment with a proactive learning attitude, cultivates the abilities and characteristics that can be practiced in life, develops innovations, and generates endless motivation (Tsai, 2011b).

“Core competency” is not only the coherence of each school system and grade level and the integration of various fields, but also emphasizes that the learning process is no longer limited to subject knowledge and skills, but should pay more attention to the combination of learning and life, and demonstrate it through practice as whole-person development (Hong and Fan, 2015). In the new curriculum teaching in Taiwan, all teachers on site know that elemental competency must be connected with life situations. The traditional text teaching method is transformed into a “life-oriented and contextualized” context, and then the learning concepts and content are brought out, emphasizing that skills and knowledge are utilizable abilities that can be utilized by individual (Ministry of Education, 2020b). Teachers at the teaching site know that they need to connect teaching with life, but it is unfamiliar for most teachers to accept the topic of “living and contextualizing” and then to bring out learning concepts and content. This level of transformation or change is relatively challenging for teachers on site. Facing the competency-oriented teaching of the new curriculum, whether teachers can fully understand its connotation and recognize it so as to produce teaching transformation is still uncertain. Teachers usually start with old experience and prior knowledge, and integrate cognition, skills, strategies, and attitudes into their teaching design. When their previous experience cannot solve the problem, they start to promote the learning motivation of thinking and solving, and transform it into teaching creativity. This study aimed to explore the teachers’ sense of identification to the new curriculum competency-oriented teaching and the relationship between the identity of competency-oriented teaching and their teaching transformation.

## 2 Literature review

### 2.1 Model theory basic theory

Teachers’ situational judgment tests (SJTs) can truly reflect students’ performance in teaching work which allow teachers to better understand their own beliefs and motivations in teaching when facing work situations and to revise their teaching strategies on a rolling basis. Secondly, there are five major aspects: class management, peer interaction, student counseling, parent-teacher communication, and teacher teaching (Chao et al., 2016; Chao, 2022). SJTs are both close to the workplace and have good predictive validity, making them increasingly popular in the past 20 years (Ou et al., 2013; Chao et al., 2016). Hence, this study used JTCS as one of the theoretical frameworks to expand the research model to help explain the correlation between teachers’ teaching attitudes, teaching willingness, and teaching identity.

Shashkin and Morris (1984) believed that “identity” can be composed of three dimensions. Firstly, belief guides the evaluation of the object, the affective component of likes and dislikes or emotional reactions; secondly, belief guides the behavioral component of the explicit behavior tendency identity of the object person, event, and objects; finally, the cognitive component includes observations, ideas, beliefs, opinions, ideas and knowledge. All of these three components are referred to as ABC identity theory. Therefore, this study adopted identity theory as one of the theoretical frameworks to expand the research model to explain the correlation between teachers’ teaching identity and teaching preparation, teaching practice, and further study.

### 2.2 Teaching attitude

Teaching attitude is significant to education scene that links towards people and incidents directly through the habitual, consistent and persistent inner psychological reaction to a certain direction and purpose through the teaching activities and process (Wu, 2000). Since the core competency cannot be taught directly, it is learned through problem-solving practices with problem events, complex situations or various difficult designs, and then the practice of strategic knowledge (Liu, 2020). Core competency is the knowledge, ability and attitude that should be possessed to adapt to the current life and face future challenges (Tsai, 2011a), and implement the curriculum practice with students as the main body (Ministry of Education, 2014a). In the learning process, the core competency is accumulated and developed continuously to contextualize cognition, skills, attitudes and values, and gradually build up the needed core competency. Hence, teachers’ teaching attitude becomes an important key in competency-oriented teaching.

### 2.3 Teaching intention

Teaching intention means the positive intention which is the driving force of teaching action. Bruner (1996) pointed out that no educational reform can take off without teachers’ active participation. Based on the knowledge theory of constructivism, learners develop through task execution, adjustment, and accumulation in specific situations (Beckett, 2008). Through repeated actions, reflections, and adjustments, they gradually deepen their inner knowledge and self-cultivation, which cannot be directly taught or instilled (Illeris, 2009). Teachers are the guides and facilitators of students’ learning, and students’ competency can be developed through contextualized learning situation guidance, delivery of tasks, methods and strategies, thinking and discussion, reflection and adjustment through teaching willingness (Wu et al., 2016). Based on this, teachers’ intention to teach has become an important factor affecting competency-oriented teaching.

### 2.4 Teacher’s sense of identity

Teacher identity refers to Eisner’s (1965) statement that four main cognitive behaviors are contemplation, inquiry, appreciation and construction. Teachers’ receptivity refers to teachers’ positive attitudes towards and behavioral intentions regarding curriculum reform (Lee and Yin, 2005). Punch and McAtee (1979) pointed out that teachers’ attitude towards educational change, participation intention and knowledge are the three main factors affecting their sense of identity. Vallacher and Wegner (1987) examined individuals’ sense of identity with vision goals from a cognitive perspective, and proposed that there is a broader range of thinking about behavioral performance, as well as a variety of methods and perspectives to explain behaviors such as imagination, awareness, and understanding. Quantitative and qualitative methods were used to analyze teachers’ identification with curriculum transformation (Waugh and Godfrey, 1993, 1995). The role of the teacher is to guide and assist students (Wu, 2017). High identity has a positive effect on the stability of individual behavior maintenance. Individuals with higher identity have more stable behavior maintenance and are less likely to change. Therefore, teachers should be involved in guiding the process of change, because teachers are the ultimate medium of change. In fact, teachers are the pioneers of change in the teaching scene (Zhang, 2014). In sum, the sense of identity not only produces meaning and value in terms of cognition, attitude, and perception, but also shows support and attitude towards individual’s behavior.

### 2.5 Teaching transformation

Teaching transformation is a transformation process in which people and society interact and are influenced by internalization and externalization. Real education is neither A for B nor A about B, but should be implemented or practiced by A with B (Fang, 2003). Transformation is through deconstructing or changing the original characteristics or attributes of something, while teaching transformation is through reflection and metacognition after teaching practice (Hong, 2018). Teaching preparation, teaching practice and further study are important basis models for cultivating self-teaching professional abilities and transforming teaching on-site (Jiang, 2021).

Teaching transformation aims to apply and practice what is learned in the classroom through the learning process of communicating and interacting with others (Cheng, 2019). Fan (2016) also believed that the practice of a competency-oriented curriculum and teaching (teaching transformation) must also master four principles: (1) Integrate knowledge, skills, and attitudes, emphasizing that learning is a complete procedure; (2) Emphasize contextualized learning more than the perception of learning meaning (making sense) and true understanding (understanding); and (3) Emphasize the learning process, strategies and methods. Curriculum planning and teaching design must combine learning content and the inquiry process in order to cultivate students’ self-learning ability and become lifelong learners; and (4) Emphasize the practice and performance so that students can integrate what they have learned, and then transfer what they have learned to real life for continuous improvement. In this study, teaching transformation was discussed from three aspects: teaching preparation, teaching practice and teacher advanced training study.

#### 2.5.1 Teaching preparation

Teaching preparation refers to unexpected things that trigger people to invest in new shocks or try to complete certain changes (Engeström, 2008). Bridges and Mitchell (2000) mentioned that teachers’ experience of psychological transformation in teaching, adjustment preparation, and the teaching site interact to influence the re-correction of practice implementation. Motivation must be induced before conversion. Reforms lead to motivation, and motivation triggers planning and teaching preparation. Murphy and Manzanares (2008) stated that teachers in the classroom adopt new technology, and contradictions arise due to the intervention of tools, which triggers teachers’ attempts to innovate and prepare for teaching in response to the new driving force. Besides, Chen and Tzeng’s (2011) study stated that many impacts and contradictions occur between old and new experiences. After gathering and reorganizing, a consensus is drawn up and a new theme is created for the course output, which will be carried out for the next on-site teaching preparation. Better teaching effectiveness can be achieved through pre-class communication and classroom discussion (Lu, 2021).

#### 2.5.2 Teaching practice

Teaching practice emphasizes the combination of learning situations and life and the ability to use practice (National Academy for Educational Research, 2014). The purpose and value of education require fundamental changes, and school curricula cannot remain unchanged (Ou and Yang, 1998). At the same time, the adjustment of teaching and learning in the teaching field and the practice of the curriculum must take into account students’ learning needs (Chan and Huang, 2020). Competency-oriented teaching integrates knowledge, ability and attitude through teachers’ teaching, focusing on situational and contextual learning and the learning process, methods and strategies, emphasizing the practice and performance of the four principles mentioned above (Hong and Fan, 2015). As the learning subject in a situation, students can utilize what they have learned and put it into practice (Yang, 2018), as teachers implement their teaching. Teachers’ competency-oriented teaching is an important issue of education for developing students’ core competencies and improving their future adaptability (Wu, 2011). Meanwhile, teachers transform their teaching through curriculum, turning ideals into practice and abstraction into concreteness (Chang et al., 2010). Teachers transform competency teaching through teaching practice at the teaching site.

#### 2.5.3 Teacher advanced training study

Teacher training research means that education is constantly facing new stimuli and challenges. Lin (2017) categorized teaching into manipulable behaviors or variables to further explore the causal relationship between teaching and learning. The depth and breadth of teacher training research are important factors in teaching design and research design (Chang, 2021). Vallacher and Wegner (1987) can distinguish the degree of identification based on the individual’s degree of understanding of the behavior. Practical teacher training researchers can have a relatively high level of identification with the object behavior and can fully understand and pay attention to the behavior. On the contrary, it is easier to think about its meaning in a one-sided way.

Hence, teachers must continue to improve their capabilities in order to analyze multiple issues in the teaching field in the future. In sum, this study aimed to explore the correlation between vocational senior high school teachers’ teaching identification and preparation, teaching practice and further study in the core competency of the new curriculum as important aspects of teaching transformation.

## 3 Research models and hypotheses

### 3.1 Research model

**Figure 1:**
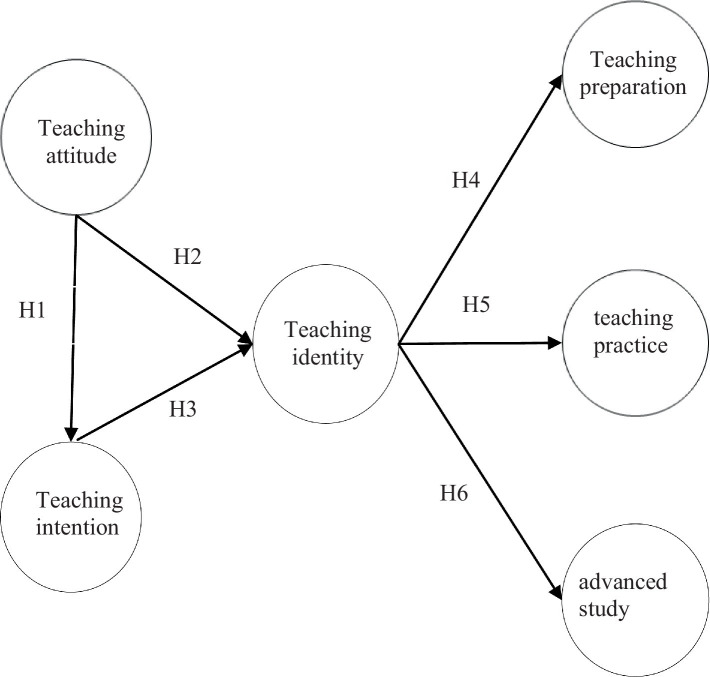
Research model.

This study adopted the ABC identity theory advocated by Shashkin and Morris, and expanded the theoretical model under the framework of SJTs, including six variables: teaching attitude, teaching intention, teaching identity, teaching preparation, teaching practice, and advanced study. A teaching transformation model was proposed to explore the relationship between competency-oriented teaching identity and teaching transformation (see Figure 1).

### 3.2 Hypotheses

Attitude refers to the positive or negative evaluation of the content of what is learned under the influence of the environment, or the action tendency of approval or opposition (Chou et al., 2020). Self’s attitude refers to the beliefs, emotions, and behavioral intentions held by individuals towards other people’s things and environments (Fan and Pan, 2021), while the behavioral component of attitude is a kind of tendency. Whether it can become a specific behavior according to the original tendency depends on the interaction between situational factors and attitudes (Wen, 2003). Teaching attitude has a direct impact on teaching behavioral intentions as called as behavioral intentions (Blackwell et al., 2001). When individuals experience something with an object, they will directly feedback their attitude towards the event and thus improve their attitude learning responses and behavioral intentions (Pieters et al., 2010; Sundar and Noseworthy, 2014).

A teacher’s teaching identity for the core competency of the new curriculum is related to whether the teaching transformation conforms to its inference or can help ensure the achievement of the teaching transformation of the core competency. Vallacher and Wegner (1987) also pointed out that the level of identity is related to action stability to some extent. Teaching preparation involves the necessary strategies according to individual satisfaction (Hossain and Quaddus, 2012). Before the transformation of teaching practice, there must be new thinking to stimulate the reform of “curriculum awareness” and implement it in teaching practice (Zhen, 2004).

When the individual’s sense of identity increases and becomes stable, transformation merges into individual independent learning, empowerment and further education. Hence, only by improving teachers’ awareness and sensitivity to curriculum reform can teachers’ interest in curriculum reform be stimulated, and then various curriculum reform concepts can be integrated into classrooms (Lin, 2019). This study proposed relevant research hypotheses of the relationships between teaching identity and teaching preparation, teaching practice and advanced study in competency-oriented teaching as follows:

*H1:* Teaching attitude is positively correlated to teaching intention.

*H2:* Teaching attitude is positively correlated to teaching identity.

*H3:* Teaching intention is positively correlated with teaching identity.

Teachers analyze teaching preparation, teaching practice, further study and teaching identity, implement competency-oriented teaching, and focus on implementation and practice (National Academy for Education Research, 2014). He (2006) believed that teachers’ teaching effectiveness should include being “able to teach,” knowing “how to teach” and having “intent to teach.” Teachers’ teaching practice belongs to the stage of “how to teach.” Allowing oneself to teach is the teaching preparation stage (Ke, 2019). The dual-track diagram of teaching and learning must start with the detection of prior knowledge, the establishment of basic knowledge structure, and the advancement of course progress (Chang, 2020).

Chen’s (1997) research on teachers’ teaching practice includes: systematic presentation of teaching materials, multiple effective teaching techniques, effective use of teaching time, establishment of harmonious teacher-student relationships, and the creation of a good class atmosphere. Teachers’ teaching practice is a continuous process (Taipei County Primary and Secondary School Principals Association, 2010). When the learning expectations of the learning subjects are completely different from the actual situation, it is doubtful whether they will continue to learn (Ngah et al., 2022). Perceptual fluency evokes pleasurable emotions (Kumar and Garg, 2010) leading to teacher preparation to teach and autonomous personal empowerment. Based on this, this study proposed the following research hypotheses on the correlation between teaching identity and teaching preparation, teaching practice, and personal development research:

*H4:* Teaching identity is positively correlated to teaching preparation.

*H5:* Teaching identity is positively correlated with teaching practice.

*H6:* Teaching identity is positively correlated with advanced study.

## 4 Research methods

### 4.1 Research process and participants

This study adopted a snowball method to collect questionnaires. Starting from October 2020, the link was distributed online to teachers of vocational high schools to fill in and pass on to other school teacher groups. A total of 769 questionnaires were received. After deleting 22 invalid questionnaires with incomplete or missing answers, there were 747 valid questionnaires, giving a 97.1% recovery rate. There were 421 questionnaires from public schools (56.4) and 326 from private schools (43.6); 325 participants were male (43.5%) and 422 were female (56.5%); 254 participants were general subject teachers (34.0%), 124 were industrial subject teachers (16.6%), 162 were commercial subject teachers (21.7%), 129 were domestic subject teachers (17.3%), and 78 (10.4%) taught other subjects.

### 4.2 Questionnaire

The content of the survey used in this study was developed and revised from past scholars’ theories related to competency-oriented teaching identity and teaching transformation. A total of 200 vocational high school teachers were recruited to pretest the questionnaire. The text content was then revised based on their feedback. The content of the questionnaire was a 5-point Likert scale, with 1 representing *strongly disagree*, 2 representing *disagree*, 3 representing *neutral*, 4 representing *agree*, and 5 representing *strongly agree* as the standard to understand the current situation of promoting the implementation of the core competency teaching of the new curriculum. Through a questionnaire survey, the study on the relationships between curriculum competency-oriented attitude and teaching intention, teaching identity and teaching preparation, teaching practice and advanced study during teaching transformation was carried out. In order to seek the construct validity of the scale, factor analysis was conducted to find the construct validity of each subscale, test the potential structure of the scale, and delete the number of questions to make each aspect more streamlined. The KMO value of the scale is 0.913, Bartlett’s sphericity test is 1595.630 (*p* < 0.01), reaching a significant level. Five vocational education experts held three expert meetings to review the content of the questionnaire before and after the administration to review the fluency and legibility of the narrative, and to correct the completeness of the facet connotation.

### 4.3 Statistical techniques

This study used Structural Equation Modeling (SEM) for empirical analysis to understand the current status of vocational high school teachers’ competency-oriented teaching, teaching identity and teaching transformation in the new curriculum and to further explore the causal relationship among them. In terms of scale reliability, validity, and the relationship between competency-oriented teaching identity and teaching transformation, stricter confirmatory factor analysis was adopted for model construction. Confirmatory factor analysis (CFA) is the measurement of a linear structural equations model, which is mainly measured by the correlations between factor loadings and factors, as a research hypothesis on the relationship between various variables or the fit between the theoretical framework and the actual sample data collected, to test whether the theoretical model can be reasonably applied to explain the sample data. In order to examine the measurement mode of each research variable, we conducted a confirmatory factor analysis among the constructs of competency-oriented teaching attitude, teaching intention, teaching identity, and competency-oriented teaching transformation shown in teaching preparation, teaching practice, and advanced study to understand the measurement structure relationships between constructs.

## 5 Research tools

### 5.1 First-order confirmatory analysis

First-order confirmatory factor analysis was used to analyze the internal validity of the model, and the original items of each construct were simplified in the CFA stage. The chi-square value (χ^2^/*df*) should be less than 5, the degree of freedom (*df*) should be less than 5, the RMSEA should be less than 0.08, the GFI and AGFI should be higher than 0.90, and items with factor loadings (FL) not higher than 0.50 should be deleted from the original questionnaire (Hair et al., 2009; Kenny et al., 2014). Based on the principle of parsimony, the number of items in each construct needed to be reduced. Therefore, the items of the teaching attitude construct were reduced from six to three; the items of the teaching intention construct were reduced from eight to five; the items of the teaching identity construct were reduced from six to four; the items of the teaching preparation construct were reduced from six to four; the items of the teaching practice construct were reduced from twelve to eight; and the advanced study construct items were reduced from six to four. The values for each construct are shown in Table 1.

**Table 1:** First-order confirmatory analysis.

Adaptability	Critical value	Teaching attitude	Teaching intention	Teaching identity	Teaching preparation	Teaching practice	Advanced study
χ^2^	–	19.732	11.362	3.866	5.031	89.611	6.167
*df*	–	5	5	2	2	20	2
χ^2^/*df*	< 5	3.946	2.272	1.933	2.516	4.481	3.083
RMSEA	<0.08	0.063	0.041	0.035	0.045	0.068	0.053
GFI	>0.90	0.989	0.994	0.997	0.997	0.970	0.996
AGFI	>0.90	0.968	0.982	0.987	0.984	0.947	0.979

### 5.2 Reliability analysis

The reliability testing standards used in this study included the combined reliability of internal consistency reliability and the Cronbach’s α test. The combined reliability of each construct must be greater than 0.70 (Fornell and Larcker, 1981), and Cronbach’s α values greater than 0.70 are preferred (Nunnally, 1978). The results of this study showed that the combined reliability of each construct was above 0.785, which is greater than 0.70, and the Cronbach’s α value of each construct was between 0.784 and 0.942, which met the standard of internal consistency reliability, as shown in Table 2.

**Table 2:** Reliability and validity analysis of items.

Item	*M*	*SD*	FL	*t* value
**Teaching attitude** *M* = 3.817, *SD* = 0.715, Cronbach’s α =0.784, CR =0.785, AVE =0.549				
1.Participating in relevant competency-oriented teaching and study activities can enhance the professional knowledge of competency-oriented teaching.	3.79	0.825	0.730	21.545
2.Formulate relevant incentives for competency-oriented teaching, which will help teachers’ intention to implement.	3.75	0.921	0.701	20.412
3.The core competency, teaching and evaluation abilities of teachers are related to the implementation of the new curriculum.	3.91	0.816	0.790	24.037
**Teaching intention** *M* = 3.990, *SD* = 0.715, Cronbach’s α =0.938, CR =0.939, AVE =0.754				
1.I am willing to discuss issues related to competency-based teaching with my peers.	4.05	0.781	0.860	22.567
2.I would like to participate in a teacher professional learning community on competency-based teaching.	3.97	0.812	0.873	22.887
3.I am willing to search for information on competency-oriented teaching on the Internet to understand the current development situation.	4.03	0.802	0.874	22.819
4.I am willing to self-teach and reflect on the process and results of competency-oriented teaching.	4.03	0.780	0.886	23.132
5.I am willing to share my experience and achievements in competency-oriented teaching.	3.87	0.816	0.849	20.730
**Teaching identity** *M* = 3.975, *SD* = 0.647, Cronbach’ s α =0.836, CR =0.819, AVE =0.533				
1.Competency-oriented teaching should not be limited to subject knowledge and skills, but should pay attention to the combination of students’ learning and life.	4.03	0.845	0.612	12.088
2.Competency-oriented teaching can cultivate students to be lifelong learners with autonomous action, communication interaction and social participation.	3.90	0.849	0.760	13.692
3.Competency-oriented teaching pays attention to the learning objectives of various fields/subjects at the same time, and can be integrated into competency teaching according to appropriate course units.	3.99	0.743	0.775	13.511
4.Core competency design should focus on learning performance (behavior, attitude, meta-cognition) and learning content (learning materials).	3.98	0.716	0.762	13.685
**Teaching preparation** *M* = 3.970, *SD* = 0.629, Cronbach’s α =0.907, CR =0.908, AVE =0.711				
1.I will incorporate the core competency design into appropriate course units.	3.99	0.679	0.795	20.689
2.My competency teaching design can connect with the context of actual life situations, so that students can learn meaningfully.	3.91	0.735	0.847	21.272
3.My competency teaching design takes into account students’ learning experience when setting learning goals.	4.01	0.699	0.875	22.185
4.My competency instructional design will emphasize student participation and active learning.	3.97	0.731	0.854	21.690
**Teaching practice** *M* = 3.848, *SD* = 0.636, Cronbach’s α =0.942, CR =0.943, AVE =0.674				
1.I can incorporate competency-oriented teaching using appropriate teaching methods.	3.91	0.699	0.795	20.714
2.The design of my course teaching situation can promote the construction and understanding of students’ core competency concepts, and help to present the key points of learning.	3.87	0.733	0.818	21.072
3.I will arrange for students to present their learning results and give immediate feedback to improve the learning of core competency.	3.78	0.799	0.805	20.650
4.I will formulate course evaluation criteria based on the competency-oriented teaching objectives.	3.77	0.761	0.838	21.253
5.I will use multiple evaluation methods (such as observation and practice) to evaluate the results of students’ core competency learning of knowledge, ability, and attitude.	3.93	0.725	0.801	20.601
6.I will design assessment questions for life situations to check the results of students’ competency learning.	3.83	0.787	0.845	21.462
7.After I teach, I observe the students’ learning outcomes to see whether the effectiveness of competency teaching is demonstrated.	3.87	0.752	0.820	20.987
8.I will evaluate students’ learning outcomes through competency-oriented teaching as a reference for the design of test questions.	3.82	0.765	0.843	21.311
**Advanced study** *M* = 3.788, *SD* = 0.726, Cronbach’s α =0.913, CR =0.915, AVE =0.729				
1.I will establish (or join) professional learning communities with my peers to discuss issues related to competency-oriented teaching.	3.78	0.824	0.889	22.959
2.I will conduct public lectures on competency teaching with my peers, communicate and learn from each other, and jointly improve our teaching effectiveness.	3.79	0.823	0.883	22.810
3.I will actively search for resources related to competency teaching (such as reading books, teaching examples, research reports, etc.) to enrich my teaching knowledge.	3.92	0.770	0.817	22.357
4.I can publicly share the teaching plan design and results of competency teaching.	3.66	0.843	0.823	21.967

***t > 3.29.

### 5.3 Validity analysis

The items of each construct were tested for external validity to diagnose the external validity of the items and discriminate the interpretable range of the research (Cor, 2016). External validity was considered significant if the *t*-value (critical ratio) was greater than 3.29 (p*** < 0.001). Table 2 showed that the *t*-value was higher than 12.09 (p*** < 0.001), which meant that all items in this study were distinctive, which could be used in this study (Green and Salkind, 2004).

The construct validity is the various measurement indicators explained by the convergent validity and discriminant validity. Based on the statistical analysis results in Table 2, the convergent validity is based on the standardized factor loading. The factor loadings of each item were between 0.612 and 0.889, which were all greater than 0.50 (Nunnally, 1978); the average extraction variation extraction values were between 0.533 and 0.754, which were greater than 0.50 (Fornell and Larcker, 1981); the combined reliability of each variable was between 0.785 and 0.943, which was greater than 0.70 (Fornell and Larcker, 1981). The AVE of each construct of discriminant validity (as shown in Table 3) should be higher than that of the other constructs (Chin, 1998); therefore, the statistical results in this study all met the test criteria and had convergent validity and discriminant validity (Byrne, 2001).

**Table 3:** Analysis of construct discriminant validity.

Construct	1	2	3	4	5	6
1.Teaching attitude	(0.741)					
2.Teaching intention	0.637	(0.868)				
3.Teaching identity	0.665	0.659	(0.730)			
4.Teaching preparation	0.562	0.730	0.663	(0.843)		
5.Teaching practice	0.590	0.697	0.622	0.822	(0.821)	
6.Advanced study	0.594	0.751	0.582	0.717	0.797	(0.854)

Chin (1998) indicated that the AVE root sign value of each construct is greater than the Pearson correlation coefficient value of other constructs, which means that the construct has discriminant validity. The analysis results show that each construct in this study had discriminant validity, as shown in Table 3.

### 5.4 Overall fitness analysis

This study used the AMOS 2.0 software for SEM analysis to test the applicability of the model and the relevance of each construct. In addition, the specifications suggested by Hair et al. (2009) are as follows: 1. Absolute fitness degree χ^2^/*df* must be less than 5, RMSEA must be less than 0.08; 2. Relative fitness degree NFI, TLI, CFI, IFI, RFI must be greater than 0.90; 3. The streamlined fit PNFI and PGFI must be greater than 0.50. Cheung and Rensvold (2002) suggested that complex factors of the model (such as the number of items and the number of factors, etc.) should not be ignored. The acceptance of GFI and AGFI greater than 0.90 used to evaluate the pros and cons of the model or whether it fits or not, appear to be too strict in complex models and too loose in simple models. This study considered the complex factors of this model, and took into account the reasoning and simplification of the model. GFI and AGFI greater than 0.80 are more likely acceptable standards. After statistical overall fitness analysis, the acceptability model constructed in this study generated the relevant index values: χ^2^/*df* = 3.282, RMSEA =0.055, GFI =0.901, AGFI =0.882, NFI =0.937, TLI =0.951, CFI =0.956, IFI =0.956, RFI =0.931, PNFI =0.848, and *PGFI* = 0.865. The above indicators all met the above-mentioned verification standards, showing that this mode was suitable for analyzing all paths constructed according to these data.

### 5.5 Research model validation

**Figure 2:**
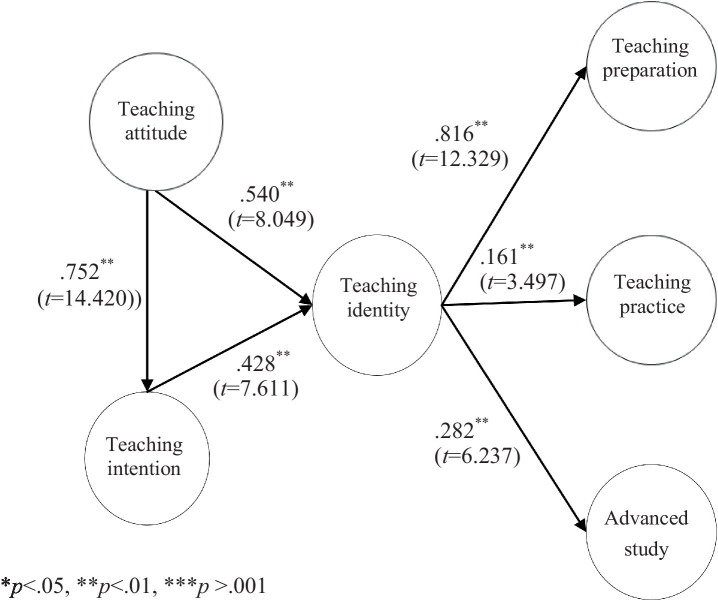
Research model validation.

The schema validation results showed: there was a positive relationship between teaching attitude and teaching intention (β=0.752**; *t* = 14.420), with explanatory power of 56.6%; there was a positive relationship between teaching attitude and teaching identity (β=0.540**; *t* = 8.049), with explanatory power of 82.2%; teaching intention had a positive relationship with teaching identity (β=0.428**; *t* = 7.611), with explanatory power of 72.1%; teaching identity had a positive relationship with teaching preparation (β=0.816**; *t* = 12.329), with explanatory power of 66.6%; teaching identity had a positive relationship with teaching practice (β=0.161**; *t* = 3.497), with explanatory power of 79.0%; teaching identity had a positive relationship with advanced study (β =0.282**; *t* = 6.327), with explanatory power of 74.6%, as shown in Figure 2.

## 6 Discussion

There is a positive correlation between teaching attitude and teaching willingness, teaching attitude and teaching identity, and teaching willingness and teaching identity.

Teaching attitude and identity: The constructivist theory of competency-oriented teaching infrastructure was developed through development rather than direct indoctrination (Illeris, 2009). This study found that among teachers with better teaching attitudes, they already possessed competency-oriented teaching abilities and actively participated in relevant competency-oriented teaching and research activities to improve their professional knowledge. Secondly, formulating relevant reward measures after competency-oriented teaching in the teaching field can also effectively improve teachers’ teaching attitude in the teaching field, which is in line with the new curriculum teaching and generates higher teaching recognition.

Teaching willingness and identity: Shashkin and Morris (1984) pointed out that all identities are constructed. This study showed that teachers with higher willingness to teach will be more able to discuss with their peers, actively participate in learning communities, actively search for relevant teaching information on the Internet, reflect after teaching, share and publish their teaching results and have a positive attitude towards teaching and generate a high sense of identity at the same time.

Teaching willingness and teaching attitude: Be aware of the intrinsic value of education and explore the meaning of life (Ministry of Education, 2020b). In competency-oriented teaching, through curriculum situations actively designed by teachers, appropriate courses are selected according to learning needs during the learning process. Learning styles, formulating learning strategies and methods, and having the courage to try innovative attitudes (Wu, 2017). Therefore, the good teaching willingness and teaching attitude of teachers in the construction teaching field are important and key factors in improving the sense of teaching identity.

There is a positive correlation between teaching identity and teaching preparation, teaching practice and further study. Sense of identity and teaching preparation: Sense of identity is the consistency of explicit behaviors and tendencies that guide people, things, and objects towards objects (Lan and Wen, 2018). Sense of identity and teaching preparation: Sense of identity is the consistency of explicit behaviors and tendencies that guide people, things, and objects towards the object (Lan and Wen, 2018). Sense of identity and teaching preparation: Sense of identity is the consistency of explicit behaviors and tendencies that guide people, things, and objects towards the object (Lan and Wen, 2018). Through teaching preparation, teachers analyze and understand the relevant teaching content, teaching materials, teaching activities, and students before teaching, so that appropriate teaching transformation can be carried out (Jiang, 2021). The study found that teachers with a high sense of teaching identity are able to integrate new courses into teaching, strive to adapt teaching courses to students’ actual life situations, and emphasize students’ participation and active learning in teaching preparation (Lin, 2018). In teaching practice, it can activate teaching activities and integrate teaching materials, and systematically and clearly provide teaching content with a complete conceptual framework; and through teaching practice, lead students to re-see the actual gap between theory and practice (Chang, 2021). This study found that those with a higher sense of teacher identity can appropriately use different learning strategies in the classroom in teaching practice, can quickly construct and understand new courses, can arrange for students to express their learning results and give feedback, and can provide multiple evaluation criteria. As well as designing different situational activities for different students’ learning styles, interactive feedback teaching is transformed into the teaching practice process.

Research on sense of identity and further study: Every time after teaching, teachers conduct teaching reflections, collaborative discussions, and through further study, feedback is provided as the basis for the next stage of teaching, so as to improve the transformation of core competencies in teaching knowledge (Jiang, 2021). The study found that teachers with a higher sense of teaching identity are able to form communities with their peers or discuss related teaching issues in teacher further education research, communicate with peers in public lectures on competency-oriented teaching, and can independently search for references related to competency-oriented teaching, teaching examples, research reports and other resources, and are willing to share their own teaching files and publication of results.

## 7 Conclusions and suggestions

### 7.1 Conclusion

Taiwan’s new curriculum emphasizes core competency to cultivate people-oriented lifelong learners; lifelong learning for all is the main driving force for sustainable development. In line with SDGS Goal 4 in high-quality education, the implementation of teaching transformation can enable teachers to better comply with the spirit of competency teaching in the new curriculum, and have a positive attitude to face the problems raised by students, and formulate more diverse and adaptive strategies. Well-trained teachers lead and transform students to acquire knowledge, skills, attitudes, and values, and to perceive a sustainable life. The theoretical and practical contributions of this study to the field of competency-oriented teaching recognition and teaching transformation are as follows: (1) the construction model of this study had a good fit, and the reliability and validity of each construct was excellent, which explained the causal relationship between the identity of competency-oriented teaching and teaching transformation; (2) sense of identity was an important trait. This study confirmed that the sense of identity in competency-oriented teaching could enhance the effect on teaching transformation and the meaning of teaching transformation at the same time; and (3) the positive correlation between the identity of the new curriculum competency-oriented teaching and the teaching transformation of the teachers working in vocational high schools was summarized as the three types of transformation trend: teaching preparation, teaching practice and further study.

First, upon the competency-teaching in the teaching field, teachers’ whose teaching attitude, teaching intentions, and high sense of identity have significant positive correlations with each other are recognized as teachers with a “foresight and a visionary predictive style” in this study. In terms of teaching preparation, teachers can actively adjust teaching strategies, have a positive attitude towards teaching motivation, and adopt multiple teaching strategies, lead students to see problems, analyze problems and then try to solve them. Students can get boost the results with less effort while learning, and their performance is remarkable at the same time.

Second, upon the competency-teaching in the teaching field, teachers’ whose teaching attitude, teaching intentions, and high sense of identity have significant positive correlations with each other are recognized as teachers with a “pragmatic teaching by example style” in this study. In the aspect of teaching practice, teachers turn knowledge into action practice, and reflect after practice before taking action. This type of teacher integrates knowledge and action at the teaching site, and is often oriented by sharing, leading students or small groups to learn by doing and learning by doing, striving for practice and rolling corrections Students or small groups can enjoy good interaction between teachers and students in this teaching environment, and the good thoughts can be cultivated or be expanded.

Last, upon the competency-teaching in the teaching field, teachers’ whose teaching attitude, teaching willingness, and high sense of identity have significant positive correlations with each other are recognized as teachers with an “empowerment-enhancing coaching style.” Teachers strive to further refine their knowledge in terms of further studies and like to explore and deconstruct problems. This type of teacher will be oriented towards interdisciplinary enhancement at the teaching site, experience different problems and solutions from the perspective of students, and then seek interdisciplinary enhancement, explore problem-solving orientation, emphasize the display of learning process results, and focus on cultivating the talent. Through changes in the campus teaching environment and competency-oriented teaching strategies, it is advocated that all learners can learn independently and hope to become lifelong learners.

### 7.2 Suggestions

To practice and act on sustainable development goals at different learning stages (Ministry of Education, 2020b), the study found that teachers’ gender, seniority, position, school type and scale, and personal and environmental factors will affect teachers’ ability to transform their sense of identity. High-ranking teachers and small-scale school teachers are more likely to accept teaching transformation.

Teaching and learning are inter-subjective processes (Chang, 2021). In competency-oriented teaching, teachers’ teaching attitude, teaching willingness, and teaching identity all have a positive impact on each other. Moreover, teaching identity also has a significant relationship among teaching preparation, teaching practice, and teacher further study in teaching transformation. In the future, it is suggested that relevant aspects can be discussed in depth to understand the interaction between competency-oriented teaching and teaching transformation. A qualitative analysis of on-site action study on additional teachers can be conducted to see whether the impact would be more significant by providing more support and training at the teaching site. Differentiated research could be conducted on in-depth academic systems or group subject teachers to obtain more results. The multi-teacher on-site information can be re-analyzed and explored from different angles and aspects through different models or theories. Finally, relevant facet questions can be expanded to increase the reliability, validity and model of the facets, adaptation.

### 7.3 Research limitations and suggestions for future research

First, this study used the snowball sampling method to conduct a questionnaire survey on teachers of technical high schools in Taiwan. Respondents may not be active and comprehensive when completing the questionnaire survey. In order to solve this problem, in future research, qualitative interviews can be added, and stratified sampling of group subjects can be expanded to investigate teachers in vocational high schools in Taiwan. This is used to repeatedly verify the relationship between the variables in this study. Secondly, this study used a cross-sectional design to collect questionnaire data, so it can only understand the participants’ feelings about the present in a specific period of time. People’s expectations will change over time, and the new curriculum will have different impacts in the early and late stages of implementation, and the benefits will mature or must be revised. Therefore, long-term follow-up should be carried out in the future to more concretely reflect the empirical evidence of the educational field, so as to be able to delve more deeply into the future educational scene and put forward strategies and suggestions for improving the competency of education. Teaching transformation of teachers’ competency-oriented teaching identity has its subjectivity and ability limitations. In order to increase the objectivity and reliability of the research results, follow-up research can also consider other objective data such as formative or summative assessments to explore the effectiveness of teaching transformation. In the end, 747 questionnaires were collected in this study, but the proportion of the study areas of the participants was not evenly distributed, and the proportions were very different. Therefore, in future research, it is recommended to expand the scope of the questionnaire to include teachers of different groups of subjects, different educational systems, and the scope of questionnaire collection, so as to reflect the spirit of the new curriculum and be more in line with the high-competency education in the competency of education. In this way, the discussion can more comprehensively explore the overall relationships among Taiwanese teachers’ competency-oriented teaching, general sense of teaching identity and teaching transformation. Under the wave of digital transformation and teaching reform, the changes in the role of teachers have also brought great challenges to them. As a result, current issues such as the relationship between teachers and students, the transformation of teachers’ teaching methods, the change of teachers’ roles, the professional characteristics of contemporary teachers, teachers’ digital literacy, teachers’ professional qualities, and teachers’ professional development all require continued attention.

## Data availability statement

The raw data supporting the conclusions of this article will be made available by the authors, without undue reservation.

## Ethics statement

Ethical review and approval was not required for the study on human participants in accordance with the local legislation and institutional requirements. Written informed consent from the patients/participants or patients/participants legal guardian/next of kin was not required to participate in this study in accordance with the national legislation and the institutional requirements.

## Author contributions

T-CL: Conceptualization, Formal analysis, Investigation, Methodology, Writing – original draft, Writing – review and editing. Y-SL: Formal analysis, Software, Writing – review & editing. J-HY: Conceptualization, Funding acquisition, Writing – review & editing, Project administration, Supervision.
