# Supplementary material for: A study on Taiwan’s vocational senior high school teachers’ teaching identity and teaching transformation when facing a new competency-based curriculum
Source: Front Psychol. 2024 Jan 31;15:1290551. doi: 10.3389/fpsyg.2024.1290551 (PMC10864582; doi:10.3389/fpsyg.2024.1290551)
Supplement: Supplementary file 1 [file Table_1.docx]

Appendix: Original questionnaire

| Construct | ◎Instructions for filling in the answers: Please put "✓" in □ and answer all the questions. | 1  Very disagree | 2  disagree | 3  average | 4  agree | 5  Very agree |
| --- | --- | --- | --- | --- | --- | --- |
| Sense of identity | 1. Core competency-oriented teaching can cultivate the knowledge, abilities and attitudes students should have to adapt to current life and face future challenges. | □ | □ | □ | □ | □ |
|  | 1. Competency-oriented teaching should not be limited to subject knowledge and skills, but should focus on the integration of students' learning and life. | □ | □ | □ | □ | □ |
|  | 1. Competency-oriented teaching can cultivate students to be lifelong learners with independent actions, communication and interaction, and social participation. | □ | □ | □ | □ | □ |
|  | 1. Core competency-oriented teaching should only be used in general fields/subjects, and professional and internship courses do not need to be integrated into core competency teaching. | □ | □ | □ | □ | □ |
|  | 1. Competency-oriented teaching not only focuses on the learning objectives of each field/discipline, but also integrates competency teaching based on appropriate curriculum units. | □ | □ | □ | □ | □ |
|  | 1. Core competency design should focus on learning performance (behavior, attitude, metacognition) and learning content (learning materials). | □ | □ | □ | □ | □ |
| Teaching attitude | 1. Core competency-oriented teaching is an important issue in global education. Teachers should keep pace with the times and keep up with educational innovation practices. | □ | □ | □ | □ | □ |
|  | 1. Core competency-oriented teaching has a positive development in the cultivation of students’ knowledge, skills and attitudes. | □ | □ | □ | □ | □ |
|  | 1. At present, core competency-oriented teaching is difficult and not easy to implement. | □ | □ | □ | □ | □ |
|  | 1. Participating in relevant competency -oriented teaching research activities can enhance the professional knowledge of competency -oriented teaching. | □ | □ | □ | □ | □ |
|  | 1. Formulate incentives related to literacy-oriented teaching to help teachers’ willingness to implement it. | □ | □ | □ | □ | □ |
|  | 1. Teachers’ core competencies, teaching and assessment abilities are related to the implementation of the new curriculum. | □ | □ | □ | □ | □ |
| Teaching willing | 1. I am willing to refer to the teaching materials or test questions of the peer core competency-oriented teaching design as a reference for teaching. | □ | □ | □ | □ | □ |
|  | 1. I am willing to discuss issues related to competency -based teaching with my peers. | □ | □ | □ | □ | □ |
|  | 1. I am willing to participate in research activities related to core competency-oriented teaching. | □ | □ | □ | □ | □ |
|  | 1. I am willing to participate in teacher professional learning communities on competency-based teaching. | □ | □ | □ | □ | □ |
|  | 1. I am willing to search for information on competency -based teaching on the Internet to understand the current development situation. | □ | □ | □ | □ | □ |
|  | 1. I am willing to self-teach and reflect on the process and results of competency -oriented teaching. | □ | □ | □ | □ | □ |
|  | 1. I am willing to share my experience and results of competency-oriented teaching. | □ | □ | □ | □ | □ |
|  | 1. I am willing to conduct public lectures on core competency teaching and communicate with peers to improve teaching. | □ | □ | □ | □ | □ |
| Teaching preparation | 1. I will incorporate the design of core competencies into appropriate course units. | □ | □ | □ | □ | □ |
|  | 1. My competency-oriented lesson plans will take into account two types of learning objectives: one is subject/field-related learning objectives, and the other is core competency projects. | □ | □ | □ | □ | □ |
|  | 1. 23. My instructional design will clearly list core competency items. | □ | □ | □ | □ | □ |
|  | 1. 24. My literacy teaching design can connect with actual life situations to make students’ learning meaningful. | □ | □ | □ | □ | □ |
|  | 1. In my literacy teaching design, students’ learning experience will be taken into consideration when setting learning goals. | □ | □ | □ | □ | □ |
|  | 1. 26. My competency instructional design will emphasize student participation and active learning. | □ | □ | □ | □ | □ |
| Teaching practice  8/12 | 1. I can use appropriate teaching methods to integrate competency-oriented teaching. | □ | □ | □ | □ | □ |
|  | 1. My teaching activities can use the connection of life situations to cultivate students' ability to solve life problems. | □ | □ | □ | □ | □ |
|  | 1. The content of my course questions can meet the core competency concepts from shallow (concrete and clear) to deep (abstract and complex). | □ | □ | □ | □ | □ |
|  | 1. The design of my course teaching situation can promote students’ construction and understanding of core literacy concepts and help to present the key points of learning. | □ | □ | □ | □ | □ |
|  | 1. I can anticipate students’ feedback on issues related to core competency-oriented teaching and have prepared countermeasures. | □ | □ | □ | □ | □ |
|  | 1. I will arrange for students to publish their learning results and provide immediate feedback to improve their core competencies. | □ | □ | □ | □ | □ |
|  | 1. I will formulate course evaluation criteria based on competency -oriented teaching objectives. | □ | □ | □ | □ | □ |
|  | 1. I will use multiple assessment methods (such as observation and implementation) to evaluate the learning outcomes of students’ core competencies of knowledge, ability, and attitude. | □ | □ | □ | □ | □ |
|  | 1. I will design assessment questions based on life situations to check students’ competency learning outcomes. | □ | □ | □ | □ | □ |
|  | 1. After I can teach, I observe students’ learning results to see if they demonstrate the effectiveness of competency teaching. | □ | □ | □ | □ | □ |
|  | 1. I will use the student learning outcomes measured through competency-based teaching as a reference for test question design. | □ | □ | □ | □ | □ |
|  | 1. I will reflect after teaching and seek solutions to problems encountered in core competency teaching. | □ | □ | □ | □ | □ |
| Further study | 1. I will participate in research activities related to competency-oriented teaching to enhance my teaching knowledge.。 | □ | □ | □ | □ | □ |
|  | 1. I will establish (or join) professional learning communities with my peers to discuss issues related to competency -based teaching. | □ | □ | □ | □ | □ |
|  | 1. I will conduct public lectures on competency teaching with my peers, communicate and discuss with each other, and jointly improve teaching effectiveness. | □ | □ | □ | □ | □ |
|  | 1. I will actively search for resources related to competency teaching (such as reading books, teaching examples, research reports, etc.) to enrich my teaching knowledge. | □ | □ | □ | □ | □ |
|  | 1. Publicly share competency teaching plan designs and results. | □ | □ | □ | □ | □ |
|  | 1. I will use the self-checklist in the relevant core competency teaching manual to plan teaching growth plans. | □ | □ | □ | □ | □ |
